# The Latest Advances in Microfluidic DLD Cell Sorting Technology: The Optimization of Channel Design

**DOI:** 10.3390/bios15020126

**Published:** 2025-02-19

**Authors:** Dan Fan, Yi Liu, Yaling Liu

**Affiliations:** 1 School of Engineering, Dali University, Dali 671003, China; fandan@stu.dali.edu.cn; 2 Precision Medicine Translational Research Center, West China Hospital, Sichuan University, Chengdu 610041, China; 3 Department of Bioengineering, Lehigh University, Bethlehem, PA 18015, USA

**Keywords:** cell sorting, microfluidics, DLD, channel design, machine learning

## Abstract

Cell sorting plays a crucial role in both medical and biological research. As a key passive sorting technique in the field of microfluidics, deterministic lateral displacement (DLD) has been widely applied to cell separation and sorting. This review aims to summarize the latest advances in the optimization of channel design for microfluidic DLD cell sorting. First, we provide an overview of the design elements of microfluidic DLD cell sorting channels, focusing on key factors that affect separation efficiency and accuracy, including channel geometry, fluid dynamics, and the interaction between cells and channel surfaces. Subsequently, we review recent innovations and progress in channel design for microfluidic DLD technology, exploring its applications in biomedical fields and its integration with machine learning. Additionally, we discuss the challenges currently faced in optimizing channel design for microfluidic DLD cell sorting. Finally, based on existing research, we make a summary and put forward prospective views on the further development of this field.

## 1. Introduction

Cell sorting plays a pivotal role in medical and biological research [1]. Traditional techniques, such as flow cytometry [2] and magnetic separation [3], have been extensively utilized in the biomedical field. Flow cytometry analyzes the physical and chemical properties of cells by passing them through a laser beam in a flowing state, integrating scattered light and fluorescence signals to provide real-time analysis [4]. In contrast, magnetic separation employs magnetic fields to isolate various substances or particles, particularly those with magnetic properties or those that can bind to magnetic materials [5,6]. However, these methods often require processing large sample volumes and may lead to inevitable sample loss during the procedure. In contrast, microfluidic technology has emerged as a significant breakthrough in the field of cell sorting due to its advantages of high precision, efficiency, low cost, and miniaturization [7,8]. By precisely controlling fluid flow within channels on the micron to nanometer scale, microfluidics enables the accurate manipulation of cells and molecular-level substances [9]. This technology has shown vast potential across various fields, including biomedicine, chemical analysis, and environmental monitoring, establishing itself as a promising new trend in cell sorting and separation.

**Figure 1:**
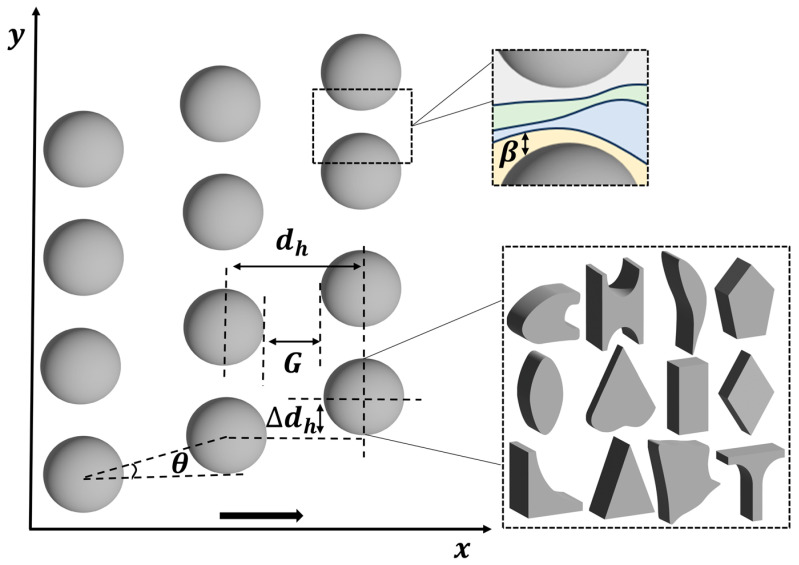
DLD geometric circular diagram. G represents the gap size, $d_{h}$ is the distance between the pillar arrays, and ${∆d}_{h}$ is the row offset. The geometric parameter ε is obtained by the ratio of ${∆d}_{h}$ to $d_{h}$. The tilt angle is given by $\theta =\tan^{-1}\left(\varepsilon \right)$. The panel in the top right shows the flow paths through a single gap (N=1/ε=4 in this case). The width β of the “first” flow channel (adjacent to the pillars) in each pillar gap provides the first-order approximation of the critical separation radius. β can typically be approximated as the minimum gap between microcolumns, which determines the size of the smallest separable particles. The bottom right corner displays various geometries of the micropillars, which include, but are not limited to, those shown in the figure. Adapted from reference [18].

Currently, microfluidic cell sorting technologies are classified into passive and active sorting based on their working principles. Active sorting utilizes external forces, such as optical tweezers [10], dielectrophoresis [11], and surface acoustic waves [12], to achieve cell sorting. In contrast, passive sorting relies on specific geometric microstructures and inherent fluid effects, such as hydrophobicity [13], inertial microfluidics [14,15], and deterministic lateral displacement (DLD) [16]. DLD, an important technology in the microfluidics field, was pioneered by Huang et al. [17] in 2004 and is primarily used to separate and sort particles of different sizes or physical properties, avoiding the issues of labeling and cell damage associated with traditional methods. In microfluidic channels, as fluid flows through arrays of regularly arranged micropillars, particles experience varying degrees of displacement due to differences in size, shape, and rigidity. As shown in Figure 1, a standard DLD array consists of a flat microfluidic channel with regular micropillars. Each row of the array is laterally displaced by a certain distance, ${∆d}_{h}$, relative to the channel flow direction (*x*-axis), generating an array tilt angle, θ, and forming a periodic flow pattern. In this pattern, the fluid is divided into multiple stagnation streamlines as it passes through each gap, creating distinct flow paths. Larger particles collide repeatedly with the micropillars, causing lateral displacement along the *y*-axis and moving along θ, thereby facilitating particle separation. Smaller particles, however, follow the fluid flow direction without undergoing lateral displacement. The motion of the particles can be classified into two modes: particles moving along the array tilt angle are in the “displacement mode” and undergo lateral displacement in each row, while particles moving in the direction of fluid flow exhibit a “zigzag pattern”. When the particle size exceeds a certain threshold, a sharp change in trajectory occurs, known as the critical diameter, Dc, which is typically smaller than the channel gap, G. Therefore, DLD technology can achieve size-based separation without clogging [18]. This technique does not rely on the chemical properties or charges of particles but instead sorts them based on physical characteristics such as size, shape, and rigidity, leading to high separation efficiency in complex samples. Compared to other separation techniques, DLD offers advantages such as high precision, label-free operation, and low cost, making it widely applicable in biological, medical, and clinical research, especially in fields such as cancer cell screening [19], blood cell sorting [20,21], and stem cell sorting [22].

In microfluidic DLD systems, an important challenge lies in the channel design, particularly the arrangement of the micropillars, which directly influences the separation efficiency and accuracy of particles. Micropillars are typically arranged in specific patterns, such as triangular, square, diamond, and other shapes [23]. These arrangements determine the separation behavior of the particles. By precisely designing the channel shape, pillar spacing, and arrangement, the separation performance can be significantly optimized. For example, studies have achieved the precise separation of particles with different sizes by adjusting the dimensions and spacing of the micropillars [24]. Over the past decade, the DLD theory has been continuously studied and refined, leading to the discovery of new DLD phenomena and the proposal of various innovative DLD designs to achieve efficient and high-throughput particle sorting [25].

Recent advances have shown that DLD can not only separate particles based on size but also perform fine classification based on other characteristics such as shape, deformability, and electrical properties. These findings further expand the application range and potential of DLD technology. To enhance separation efficiency, researchers are constantly exploring more efficient separation schemes. For example, optimizing the geometric parameters of the DLD channels, designing high-throughput parallelized channel structures, and improving the surface properties of the channels have significantly improved separation efficiency. Despite significant advancements in DLD technology, several challenges persist, including the reliance on experimental iterations for optimizing channel parameters, limited real-time screening strategies, and considerable individual variation in complex biological samples [18]. To overcome these challenges, the integration of microfluidic cell sorting technology with machine learning has emerged as a key area of research. An increasing number of researchers are proposing to combine these two approaches, envisioning the development of more convenient and accurate models. For instance, McIntyre et al. [26] introduced machine learning for microfluidic device design and control, which could have future applications. Machine learning methods can optimize DLD structural parameters, predict particle trajectories, and enable adaptive screening, thereby enhancing the sorting capability of complex biological samples. Therefore, we propose a method that utilizes machine learning to reverse-engineer the DLD geometry and fluid dynamics characteristics, aiming to overcome the limitations of traditional designs and achieve the development of more efficient and flexible microfluidic separation systems. This interdisciplinary integration has driven the development of DLD technology and opened new research directions, offering innovative possibilities for the field of cell sorting and separation.

This review aims to summarize the latest advancements in microfluidic DLD channel design in recent years. First, we will provide an overview of the design elements of microfluidic DLD cell sorting channels, focusing on the key factors that influence separation efficiency and accuracy. Next, we will discuss the latest developments in channel design for microfluidic DLD cell sorting technology and its applications in the biomedical field, as well as the challenges faced by microfluidic DLD cell sorting. Finally, based on the current research progress, we will summarize future research directions and offer our forward-looking perspectives on the development of this field.

## 2. Design Elements of Microfluidic DLD Cell Sorting Channels

Dc is a key parameter to consider when designing DLD arrays, as it determines the particle size that the array can separate and influences the channel geometry and fluid dynamics design. The geometric parameters that influence Dc are depicted in Figure 1. In a DLD device, when fluid flows through the arranged pillar structures, particles smaller than Dc follow the fluid streamlines, while particles larger than Dc undergo lateral displacement. Therefore, accurate calculation of Dc is crucial to the design of the DLD system. Early on, Inglis et al. [27] developed a theoretical model to determine Dc by assuming a parabolic velocity profile at the inlet of the DLD unit, providing a practical structural design theory for cylindrical DLD systems. The critical diameter model calculation is as follows:

(1) $$D_{c}=G\left(1+2w+\frac{1}{2w}\right)$$

(2) $$w={\left[\frac{1}{8}-\frac{\varepsilon }{4}+\sqrt{\frac{\varepsilon }{16}\left(\varepsilon -1\right)}\right]}^{\frac{1}{3}}\left(-\frac{1}{2}-\frac{\sqrt{3}}{2}i\right)$$

Here, G denotes the gap between the pillars, representing the minimum width of the fluid passage between them. ε represents the ratio of the distance each row moves horizontally to the gap. This method has remained efficient in many studies in recent years and provides a good approximation of actual steady-state conditions. Notably, subsequent researchers have made several modifications and optimizations to this approach, further enhancing its applicability [28,29]. Kottmeier et al. [30] proposed that when $R_{e}\leq 1$,

(3) $$D_{c}=1.4.G.\varepsilon^{0.48}$$

This formula, derived through experimental fitting, is more suitable for practical microfluidic DLD devices. Compared to the Inglis model, this method is more intuitive and exhibits greater applicability in real DLD systems.

**Figure 2:**
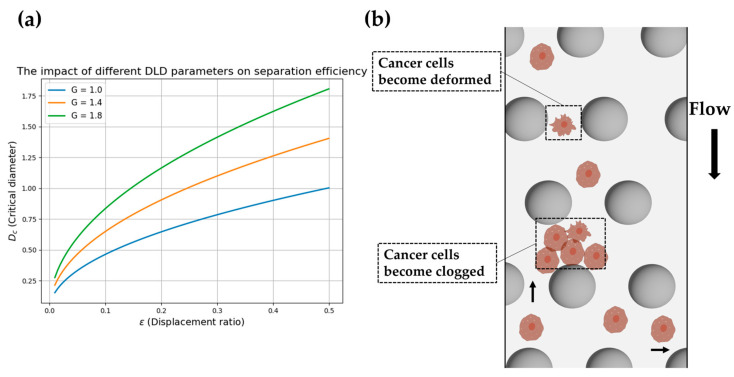
The trend of Dc as a function of ε and the behavior of soft particle channels. (**a**) The effect of different DLD structural parameters (G values) on separation efficiency, along with the variation in Dc with respect to ε. Curves in different colors represent distinct G values, corresponding to various DLD design configurations. (**b**) The interactions of cancer cells with the channel are described, showing how cancer cells deform, accumulate, change direction, and move along different streamlines beneath the array when the flow rate changes.

From the model, it can be observed that Dc is primarily influenced by G and ε, as illustrated in Figure 2a. When G is large (green curve), Dc increases, indicating that larger particles can be separated. In contrast, when G is small (blue curve), Dc decreases, making the DLD system more suitable for separating smaller particles. As ε increases, Dc increases, enabling the DLD device to separate larger particles.

### 2.1. Channel Geometry Design

Channel geometry plays a crucial role in microfluidic DLD cell sorting. Based on the design principles of DLD technology, several geometric parameters must be considered when developing a DLD array with a specific critical diameter. These parameters include the channel width and height, the spacing between the pillars, and the diameter of the pillars. In 2019, an excellent review was published that thoroughly explored the geometric factors influencing the critical diameter, particularly focusing on the impact of pillar design and pillar arrangement on DLD separation efficiency [25]. Table 1 provides a detailed summary of the various factors in channel geometry that affect particle separation.

In an ideal scenario, a wider channel allows more particles to flow, while a narrower channel provides more lateral displacement. An increase in channel height typically results in a larger critical diameter, and as the channel height increases, the separation efficiency also improves [32]. Increasing the channel length generally provides a longer particle flow time [33], thereby increasing the opportunity for particles to interact with the micropillars. However, excessively long channels may lead to a decrease in flow velocity, which increases separation time and reduces overall separation efficiency. Therefore, it is essential to find a balance between channel length and separation efficiency during the design process.

The size and spacing of the pillars are further key factors affecting particle displacement. Research by Zeming et al. [21] demonstrated that by arranging wider pillar gaps laterally and gradually decreasing the gap along the flow direction, better separation performance could be achieved while maintaining a high throughput. Smaller pillar gaps result in stronger lateral forces on the particles during flow, causing larger displacements and enabling separation based on size. In contrast, larger pillar gaps allow particles to flow more easily along the main flow direction, reducing lateral displacement. Furthermore, studies have shown that larger pillar sizes cause streamlines to tilt, thereby increasing lateral displacement and altering the “zigzag” flow pattern [34].

Different micropillar geometries also significantly affect cell separation. For example, pillar shapes such as triangular, rectangular, and circular ones offer distinct advantages depending on the application. Smaller critical diameters are often beneficial for microfluidic devices, such as reducing clogging effects [35]. Some studies have shown that triangular pillars can reduce the critical diameter [31]. Another study developed a microfluidic chip based on a micropillar array (MPA-Chip), which includes multiple shapes of micropillars, such as rhomboid, rectangular, circular, and triangular ones [39]. Rectangular micropillars are commonly chosen for their simple fabrication and stable structure, making them especially effective for separating larger particles. Sun et al. [40] demonstrated a micropillar particle separator fabricated using optical projection lithography based on digital micromirror devices (DMDs), which included cylindrical, rectangular, and triangular arrays, effectively separating 20 μM and 200 μM polystyrene microspheres. Loutherback et al. [41] proposed a theoretical model and experimental data suggesting that future designs for deterministic lateral displacement arrays should consider using triangular pillars, and they also explored the effects of vertex rounding, pillar size, and shape in practical designs. Trapezoidal designs are particularly suitable for separating smaller particles, especially in complex cell sorting applications. Circular or rounded-edge micropillars help reduce dead zones and local flow instability in the fluid, minimizing particle retention, and are especially suitable for separating irregularly shaped or deformable particles, such as deformed cells and non-spherical cells [42]. Additionally, other studies have summarized the advantages and disadvantages of different geometric shapes [36].

The layout of the micropillars (e.g., linear arrangement, staggered arrangement) also directly influences the particle flow path. Staggered layouts increase the opportunity for particles to interact with lateral displacement and flow paths, thus enhancing separation precision [37]. Chen et al. [43] studied two-dimensional fluid flow in microfluidic devices with different micropillar designs and found that, in staggered arrangements, the fluid flowed not only through the center of the array but also around the pillars, which led to a more uniform distribution of fluid compared to aligned pillar arrays, improving separation efficiency.

The curvature of the channel (i.e., the degree of channel bending) also affects particle separation. The design of the curvature alters the fluid flow characteristics, which in turn affects the particle motion trajectory [38]. Appropriate curvature can promote the lateral displacement of particles, particularly for smaller particles. Excessive curvature may lead to unstable fluid flow and negatively impact separation, so curvature design requires precise control.

### 2.2. Fluid Dynamics’ Design

In passive microfluidics and label-free microfluidic applications, computational fluid dynamics (CFD) is the most commonly used numerical tool. CFD simulations can be performed using various software tools such as ANSYS Fluent 17.0 [44], COMSOL Multiphysics 5.4 [45], and OpenFOAM [46]. These tools assist in analyzing how fluid velocity, shear force, and other fluid dynamics parameters influence cell flow and separation efficiency. Additionally, the viscosity of different liquids significantly affects cell behavior and separation performance, which must be considered during fluid dynamics’ design.

Flow velocity is a crucial parameter that determines the speed of fluid movement and the relative motion of particles [36]. Higher flow velocities cause particles to move quickly along the main direction of the fluid, reducing their interaction with micropillars and suppressing lateral displacement, which ultimately decreases separation efficiency. High flow velocity increases the effective diameter of the particles, causing their trajectories to focus on the center of the channel. In contrast, lower flow velocity favors precise separation as the particle movement is more controlled by the micropillars. Studies have shown that the separation efficiency of cancer cells is closely related to the magnitude of flow velocity. [47]. Both excessively high and low flow velocities can negatively impact separation performance. Therefore, the precise control of flow velocity is necessary to ensure that it is within the optimal range for particle separation.

In microfluidic channels, shear force affects particles depending on their size, shape, and rigidity. Different types of cells exhibit varying sensitivities to shear force, and cells exposed to high shear may be damaged or deformed, which can affect their biological activity. As flow velocity increases, shear force also increases, and particle deformation becomes an increasingly important issue, complicating the dynamic behavior of particles in the system [48]. The particle Reynolds number (ReP) indicates that at moderate channel Reynolds numbers, shear forces and wall lift have minimal effects on particles in DLD devices. Thus, shear force must be controlled in DLD system design to avoid unnecessary cell damage. For flexible cells (such as red blood cells or certain cancer cells), excessive shear forces can cause deformation or rupture. Proper shear forces facilitate the movement of cells along the main flow direction, while lower shear forces ensure that cells are not damaged by excessive pressure. In DLD separation, lower flow velocities typically reduce the impact of shear forces and decrease cell damage.

In high-flow or large-scale DLD devices (such as those with millimeter-sized gap dimensions), the Reynolds number ($R_{e}$) of the fluid may exceed 1. Studies have shown that when $R_{e}$ exceeds 1, particles are influenced by inertial lift, which can alter their trajectories and affect separation efficiency [49]. However, optimizing the separation performance using inertial lift or avoiding unwanted inertial effects under high throughput conditions remains an active area of research [10].

The viscosity of the liquid also significantly affects cell behavior. Studies have shown that viscoelastic fluids can achieve the dynamic control of particle separation through shear-thinning effects [50]. Previous research has been limited to Newtonian fluids, but Li et al. [51] proposed that using viscoelastic fluids in DLD arrays can provide the elastic modulation of particle separation. The results indicated that viscoelastic fluids not only regulate shear-thinning effects but can also dynamically adjust the critical diameter by altering flow velocity. Higher liquid viscosity increases internal friction during fluid flow, thereby enhancing interactions between particles and the fluid, which slows the particle movement. In DLD technology, high-viscosity liquids assist in precise cell separation by prolonging the collision time between particles and micropillars, promoting lateral displacement. However, excessively high viscosity may cause flow instability, especially in narrower channels, leading to flow dead zones or local flow irregularities, which can affect separation performance. Moreover, high-viscosity fluids may increase system complexity, requiring higher pressure and energy consumption. In contrast, low-viscosity liquids flow more smoothly, and particles move faster. However, this can reduce the interaction time between particles and micropillars. Additionally, particles in low-viscosity fluids have difficulty undergoing effective lateral displacement, leading to lower separation efficiency.

In microfluidic DLD systems, fluid is typically required to maintain laminar flow [52]. Earlier studies focused on using laminar flow for separation at low Reynolds numbers. Laminar flow exhibits uniform streamlines and predictable separation characteristics but relies on lower flow velocities and may fail if the velocity is too high [53]. In contrast, in turbulent flow, the fluid streamlines become irregular, and the velocity distribution becomes uneven, making particle trajectories unstable. High-viscosity liquids help maintain laminar flow, while low-viscosity liquids are prone to turbulence. Ensuring that the fluid remains in laminar flow is crucial for stable separation, so selecting appropriate viscosity and flow velocity to maintain laminar flow is key to enhancing separation efficiency in DLD systems.

### 2.3. The Interaction Between Cells and the Channel

The rigidity, morphology, and surface properties of cells significantly affect their behavior in DLD channels [23]. Rigid, hard spherical particles typically travel along one of two possible paths in the microfabricated pillar array, depending on their size. However, the situation is more complex for soft particles. Properties such as size and deformability influence the clogging and fouling of the channel (see Figure 2b). There are several unique challenges when processing soft biological cells in microfluidic DLD devices. Due to the flexibility of the cells, they can deform in response to forces within the array [54]. For instance, Zhang et al. [55] found that sharp geometric obstacles in microfluidics can promote the sorting of deformable cells. Additionally, the viscosity of the separation buffer affects the membrane deformation dynamics at different positions within the DLD array. Studies have shown that under varying viscosity conditions, the trajectory patterns of red blood cells may exhibit positive, negative, or neutral zigzag and negative transport modes [56]. Consequently, the apparent size of the cells varies with changes in the flow rate within the array, and under greater forces, cells may adhere to the surface. Moreover, some cells are not spherical, and their orientation influences their path through the device, potentially causing them to shift toward different flow lines beneath the array or even cause blockages within the channel.

When cells are more rigid, they are typically harder to deform and may become obstructed in smaller channels or deviate from their expected flow paths. In contrast, more flattened or irregularly shaped cells may exhibit different migration behaviors, potentially being captured or redirected. For deformable, non-spherical particles, their behavior leads to more complex movements [57]. Due to random Brownian motion, smaller particles tend to interact less with the array, while larger particles may collide with the pillars and experience slight diffusion effects [58]. The surface characteristics of cells, such as surface charge [59,60,61], hydrophilicity, or hydrophobicity [62,63], also affect the interaction between cells and the fluid, which in turn influences the deflection pattern and sorting efficiency of cells in the DLD channel.

## 3. Recent Advances in Microfluidic DLD Channel Design

### 3.1. Optimization and Innovation of Geometric Parameters

#### 3.1.1. New Columnar Geometric Design

Recently, new columnar geometric designs have demonstrated a unique trend in research. Exploring innovative columnar shapes, arrangements, and other methods to enhance separation accuracy is crucial. Traditional cylindrical columns have been modified into various columnar shapes, including triangular ones [41], airfoil ones [64], I-shaped ones [65,66], L-shaped ones and their variants [67], asymmetric shapes [68], and optimized shapes [35].

**Figure 3:**
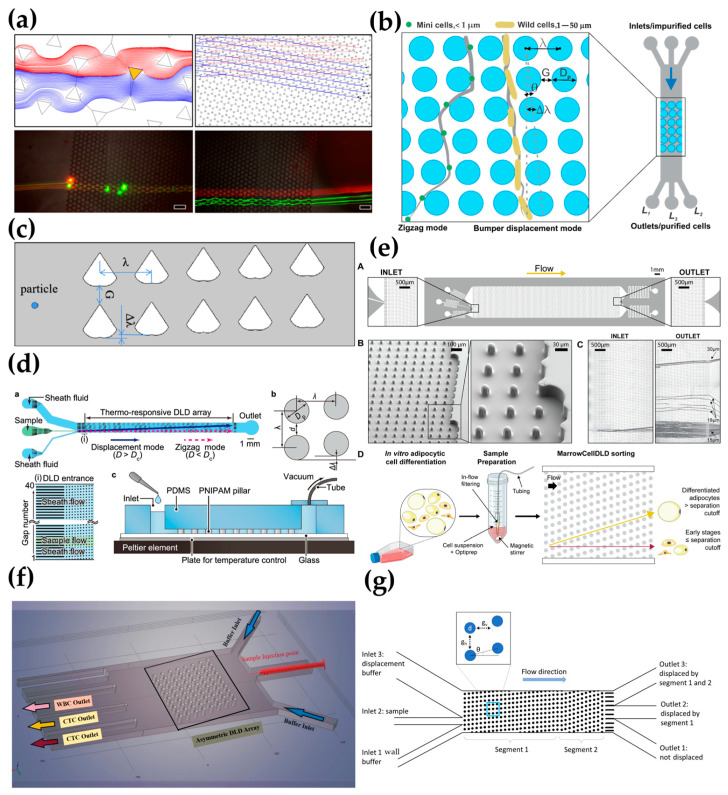
(**a**) A hexagonal array triangle (HAT) geometry and working diagram [69]. (**b**) A schematic of the DLD microchip for separating E. coli minicells (spherical) and parental (rod-shaped) cells [70]. (**c**) A computational domain [71]. (**d**) A thermally tunable microfluidic DLD device. **a** Schematic illustration of the thermo-responsive DLD device. The inset illustrates the entrance of the DLD array, and each gap between the pillars is numbered from 1 to 40. **b** A rhombic unit cell with the array parameters. Dp = 20 μm, λ  = 50 μm, Δλ/λ = 0.05, and d = λ − Dp = 30 μm. **c** Schematic side view of the device [75]. (**e**) A MarrowCellDLD device and its operation. **A** MarrowCellDLD chip: insights on inlet and outlet regions terminated with 10 rows of straight pillars respectively before and after the MarrowCellDLD array active region (tilted array). **B** Scanning electron microscopy images of the MarrowCellDLD array chip with a 19 μm separation cutoff (critical size). **C** Inlet and outlet trajectories of polystyrene microbeads of 15, 18, and 20 μm in size transiting a MarrowCellDLD chip with 19 μm critical size. **D** Experimental workflow to sort differentiated adipocytes by MarrowCellDLD [76]. (**f**) A 3D view of the modeled microfluidic device [77]. (**g**) A schematic illustration of a two-segmented DLD post-array for particle fractionation [78].

Razaulla et al. [69] investigated a novel patterned DLD geometry called the hexagonal array triangle (HAT) geometry, as shown in Figure 3a. This geometry was achieved through the self-assembly of a monolayer of nanospheres. Finite element simulations were used to characterize the DLD separation performance of the HAT structure. The results revealed that when the array angle was less than 7°, the HAT structure exhibited better particle sorting ability (i.e., smaller critical diameter-to-gap distance ratio) compared to previously published, circular-column, parallelogram-type DLD arrays. To further optimize geometric design parameters, Sherbaz et al. [70] proposed a DLD microchip (see Figure 3b) that can separate rod-shaped bacterial cells up to 10 μM from submicron spherical cells. Furthermore, they optimized geometric parameters, including the column shape, size, angle, critical radius, channel width and depth, and number of arrays, achieving a single-stage separation efficiency of up to 75.5%. These findings lay the foundation for the development of high-throughput separation and purification modules, which could potentially be directly integrated into bioreactors in the future. Chen et al. [71] designed a novel inverted heart-shaped microcolumn (see Figure 3c), which has a smaller critical size and simulated particle trajectories under a bidirectional coupling condition. By simulating the separation process of two types of cells, they successfully separated white blood cells and red blood cells. Additionally, some studies showed that a DLD separation chip with topologically optimized pillars demonstrated high separation efficiency in numerical simulations and microbead and cell experiments [72]. Another study achieved approximately a ten-fold reduction in the inherent critical diameter of the device by optimizing asymmetric micron-sized columns and gap arrays, coupled with an alternating electric field perpendicular to the fluid flow [73]. Marhenke et al. [74] designed a device with 4 μM pillar spacing and 50 periods. During fabrication, SU-8 masters with different heights (15 and 30 μM) were used for photolithography to form vertical sidewall structures. They studied the effect of flow rate on the separation efficiency of 0.45 and 0.97 μM particles. The results showed that in a 30 μM-high device, the replacement efficiency of the 0.97 μM particles exceeded 99%, while in a 15 μM-high device, the efficiency ranged from 46% to 57%.

Tottori et al. [75] proposed a novel DLD technology utilizing thermoresponsive hydrogels (poly(N-isopropylacrylamide), PNIPAM) as posts to flexibly adjust the critical diameter value, as shown in Figure 3d. By tuning the Dc value, they achieved a “switching” operation for particle separation, successfully separating 7 μM and 2 μM beads. In May 2023, they introduced a new high-efficiency microfluidic chip based on micropillar arrays, called MPA-Chip. This chip features micropillar arrays with various geometries (diamond, rectangular, circular, and triangular). Numerical simulations showed that the diamond geometry was the most effective and optimized micropillar design for circulating tumor cell separation [39]. Another study proposed a new method for manufacturing a tunable DLD chip by altering the flow line direction. This chip was easy to fabricate and allowed flexible performance adjustment by modifying two control parameters [79]. Additionally, a specialized microfluidic method for label-free sorting of fragile bone marrow-derived cells (MarrowCellDLD) was developed, as shown in Figure 3e. This method, designed for sorting large and fragile cells from bone marrow cultures, demonstrated significant potential for application [76].

Bhattacharjee et al. [77] successfully simulated an asymmetric microfluidic device based on a DLD array using COMSOL Multiphysics 5.4 software. The model employed an asymmetric DLD array layout with cylindrical micropillars (see Figure 3f). The simulation study investigated the effect of the sample inlet release angle on the trajectories of circulating tumor cells (CTCs) and white blood cells (WBCs). The results indicated that a clockwise-tilted sample inlet produced better separation results compared to a counterclockwise-tilted inlet. In the same year, Kottmeier et al. [78] developed an optimized process using deep reactive ion etching on silicon and the anodic bonding of glass covers to create pressure-resistant arrays, successfully achieving a 120 μM depth and 23 μM pillar gap, with a length-to-width ratio of 6:1, as shown in Figure 3g. At the highest flow rate, this device successfully separated suspensions containing mixtures of 2 μM and 10 μM particles, as well as 5 μM and 10 μM particles. The study demonstrated that a higher aspect ratio helped improve the throughput of the DLD device without compromising its selectivity.

Through the optimization of geometrical structures (such as hexagonal-arranged triangle geometries and heart-shaped micropillar designs), the use of novel materials (such as thermoresponsive hydrogels), and process improvements, researchers have enhanced the separation efficiency and performance of DLD devices. These geometric shapes significantly influence fluid dynamics’ characteristics. Geometries such as hexagonal and heart-shaped micropillars primarily affect fluid dynamics in terms of flow patterns, fluid resistance, vortex formation, and mixing efficiency. These structures regulate fluid behavior by altering the flow path, generating vortices, and influencing pressure distribution, thereby playing a pivotal role in microfluidic technology, biomedical applications, and engineering. For instance, the hexagonally arranged triangular geometry has been validated through finite element simulations, demonstrating its advantages in fluid flow. Compared to traditional cylindrical pillar arrays, it enhances particle sorting capability and exhibits a lower critical diameter-to-gap spacing ratio [69]. In contrast to square arrays, the fluctuations in the hexagonal array manifest as an unordered zigzag pattern at the device scale, with smaller fluctuation sizes. The vortices and flow patterns induced by the hexagonal structure in the fluid are more complex, leading to a lower friction coefficient and reduced wear during fluid transmission. Furthermore, the vortex-induced resonance response of the hexagonal pillars does not occur at specific frequencies, and the deviation increases as the mass ratio decreases. In another study, the heart-shaped micropillar design effectively influenced the lateral displacement of particles due to its unique geometry [71]. The special shape of heart-shaped micropillars generates complex local flow fields around them. This shape promotes vortex formation in the fluid, enhancing mixing efficiency and improving mass transfer. Additionally, the shape of heart-shaped micropillars leads to uneven pressure distribution near the micropillar surface. The curved sections increase fluid resistance, while the pointed sections may create localized low-pressure zones. This uneven pressure distribution impacts the overall fluid flow characteristics. Numerical simulations of thermo-responsive hydrogel pillar designs have demonstrated that by adjusting the stiffness and size variations in the hydrogel pillars, the critical separation size can be dynamically tuned to accommodate the separation of particles of varying sizes. These studies have broadened the application range of DLD technology, including cancer cell separation, the sorting of fragile bone marrow-derived cells, and the development of high-throughput separation modules. By integrating experimental and simulation data, researchers can accurately evaluate the fluid dynamics characteristics of each geometric design. These innovations not only advance the application of DLD technology in laboratory settings but also provide theoretical support and technical foundations for future integration into practical applications, such as bioreactors. Through the continuous optimization of geometric structures, materials, and processes, the full potential of DLD technology in practical applications will be further realized.

#### 3.1.2. Multifunctional DLD Channels

The integration of multiple separation stages or the adoption of modular designs has shown significant advantages in the construction of DLD channels, and the importance of this design approach is self-evident. By breaking down complex systems into independent, manageable modules, we can more effectively develop and maintain each component. Each module can be independently optimized and upgraded, thereby enhancing the overall flexibility and scalability of the system. Therefore, integrating multiple separation stages or adopting modular designs is not only a technological trend but also a key strategy to ensure that DLD channels remain competitive in a rapidly evolving technological landscape.

**Figure 4:**
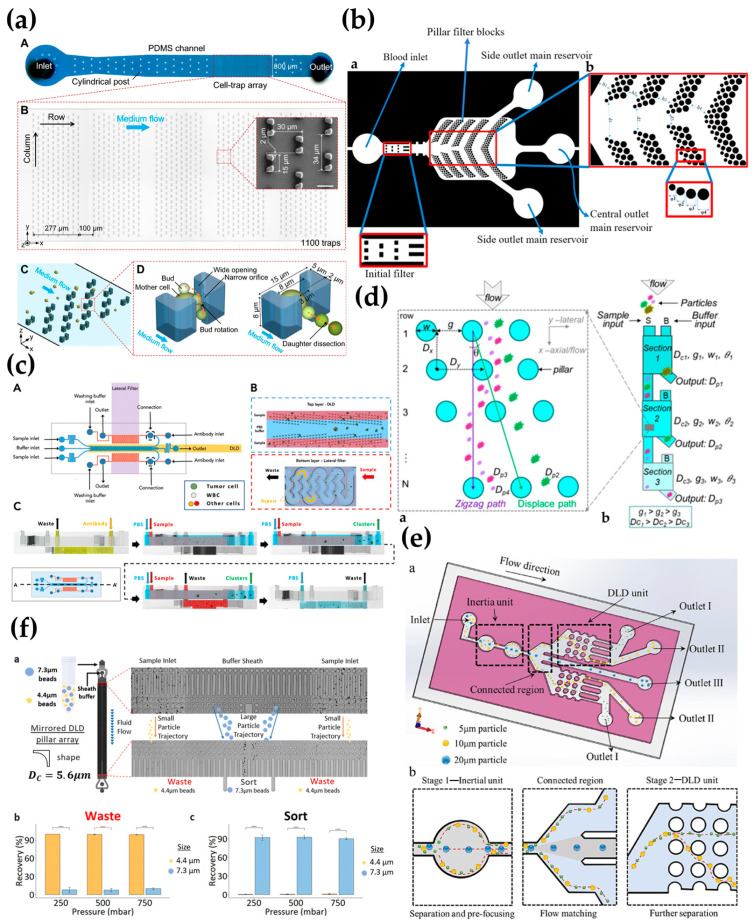
(**a**) An overview of the microfluidic DYLC chip. **A** Photograph of the PDMS microchannel including 53 cylindrical posts and a cell-trap array. **B** Micrograph of the array patterning into 5 subarrays with a gap of 100 μm in between. **C** A schematic cartoon showing the cell loading and trapping processes in the array. **D** 3D schematics showing the geometric dimension of the “leaky bowl”-shaped trap, the hydrodynamic bud rotation and the concerted daughter dissection [80]. (**b**) A magnified view of the components of the microchip. **a** As it can be seen, the microchip structure includes an entrance enclosure, initial filters, a main reservoir, a central outlet reservoir, and side outlet reservoirs. **b** φ1, φ2, φ3 and φ4 are defined as the pillar diameters in each row. h1, h2, h3, and h4 are defined as the horizontal distance between the blocks and d1, d2, and d3 are defined as the vertical distance between the blocks [81]. (**c**) A top view and the functionality of the dual-layer microfluidic biochip. **A** The top view and functions of the two-layer microfluidic biochip. The chip consists of two layers with different functional modules. **B** Here shows the schematic diagram of the function of the upstream DLD cluster-sorting module in the top-layer channel (the blue-dashed-rectangle part) and the downstream antibody-coating lateral filter module in the bottom-layer channels (the red-dashed-rectangle part) of the microsystem chip. The working process of the two-layer microfluidic biochip with the side-view section drawing is shown in **C**. Line A to A ′ 2 is the section line of the two-layer microfluidic biochip [83]. (**d**) A schematic diagram of the DLD separation column array and conceptual diagram of the external balanced multi-size DLD separator. **a** An illustration of the pillar array for DLD separation showing key model parameters, and **b** a concept diagram for the externally balanced multi-size DLD separator with multiple cascade gap-scaled sections [84]. (**e**) A schematic illustration and separation mechanism diagram of the two-stage separation platform. **a** Schematic and **b** separation mechanism of the two-stage separation platform. The platform is composed of an inertial unit consisting of con traction–expansion arrays, two DLD units consisting of micropillar arrays and a connecting region of the two. Particles are initially separated and prefocused in the first-stage inertial unit. Flow rates are redistributed within the connecting region through flow matching. The second-stage DLD units are utilized to further separate medium and small particles [85]. (**f**) A characterization of the DLD sorting device. **a** Sorting characterization of the DLD device using 4.4 μm and 7.3 μm beads. **b** Graph showing the bead recovery in both the waste channels. **c** Graph showing the bead recovery in the sort channel. Paired two-tailed Student *t*-test was used to determine the statistical differences between outputs [86].

Wang et al. [80] developed a high-throughput microfluidic chip for long-term diploid yeast culture (DYLC), optical detection, and cell aging analysis (see Figure 4a). The DYLC chip contained 1100 “leak-bowl”-shaped traps arranged in an array to capture single cells under laminar flow conditions and effectively removed progeny cells via hydrodynamic shear forces. By simulating the key parameters of the traps and array geometry through computational fluid dynamics and experimental characterization, the capture efficiency and long-term maintenance of single cells were optimized. The results showed that the microfluidic DYLC chip enables the high-throughput capture, reliable maintenance, and long-term culture of diploid yeast cells and can be used for RLS measurements and dynamic morphology analysis in yeast aging studies. Darboui et al. [81] designed a microfluidic chip inspired by the structure of fish gills (see Figure 4b), which uses DLD technology to separate blood cells with columns of varying distances and diameters. The chip’s main reservoir is flanked by filtering blocks, optimized with one inlet, three outlets, and ten filtering blocks, achieving a separation efficiency of 98%. Additionally, a nanoscale DLD array with gap sizes ranging from 25 to 235 nm has been demonstrated to have potential for sorting and quantifying nanoscale biocolloids [82]. Recent studies also show that DLD technology can separate particles into multiple groups of different sizes by internally cascading fluidic balance sections with varying geometric parameters.

Li et al. [87] proposed a new method that combines a contraction–expansion array (CEA) channel with a DLD array. This approach first uses the combined effects of inertial and Dean resistance forces to achieve particle focusing and preliminary separation in the CEA channel. Subsequently, after passing through the DLD array, the trajectory of the particles is adjusted by the periodic arrangement of the continuous flow columns, which directs particles of different sizes to different outlets. Experimental results showed that the coupled design of a triangular microcolumn DLD array could further enhance separation purity and efficiency. With 5 μM polystyrene microspheres as the target particles, the separation efficiency exceeded 99%, with a separation purity of 96.1% at high flow rates. Despite the advantages of the CEA channel in particle velocity and separation purity, it required additional sheath flow to achieve particle focusing, and the overall sorting purity did not show significant improvement. Lee et al. [88] developed a scalable microfluidic cascade sorter that combines the benefits of a spiral inertial sorter and a DLD sorter for the high-throughput, label-free enrichment of mesenchymal stem cells (ADSCs) prepared from human adipose tissue. The microfluidic sorter consists of a spiral inertial sorter and a DLD sorter, which separate cells based on size differences. Cell counting results showed a separation efficiency of 90%, and the ADSC subpopulations enriched by the microfluidic cascade sorter exhibited a six-fold increase in amplification capability in the tissue culture, offering new possibilities for generating transplant-scale stem cell products. The study also proposed a two-stage microfluidic chip (see Figure 4c) designed for cancer research and clinical applications to study CTC clusters. The chip integrates a microfluidic biological chip in which the first-stage sorting mechanism in the top channel is based on the object size, coupled with a series of DLD arrays to achieve efficient sorting [83].

Yin et al. [84] introduced a new, external, balanced, multi-section cascade DLD method (see Figure 4d) and developed a detailed model to define the interactive design parameters for this method. By applying manufacturing size constraints to this model, the study found significant impacts on the simulation results of the device length, further narrowing the available design space. The study also highlighted the design results of circular and I-shaped pillars, emphasizing the impact of different design choices on separation performance. The results showed that by adjusting the gap size and critical separation size in multiple sections of the cascade, the separation of particles in different size ranges and higher size resolution could be achieved. Additionally, a numerical study was conducted on a size-dependent cascade inertial sorting and DLD microfluidic device for the sheathless separation of tumor cells. The cascade structure eliminated the need for additional force fields, reducing the device complexity, simplifying operations, and minimizing sample contamination opportunities [89]. Another study presented a three-stage inertial method comprising a rectangular channel, a contraction–expansion array (CEA), a lateral separation channel, and another rectangular channel. This method successfully separated 20 μM particles from a mixture containing both 10 μM and 20 μM particles, with extremely high precision. The technique achieved a 100% separation efficiency and a separation purity of 96.1% [90]. Lu et al. [85] proposed a novel two-stage separation platform that combines DLD technology with inertial sorting (see Figure 4e). The first-stage inertial unit consists of a contraction–expansion array to separate large particles and pre-focus the remaining particles. In the second stage, a DLD device further separates medium and small particles. Experimental results demonstrated that by combining the inertial and DLD units, the efficient (96.3%, 94.7%, and 100%) and high-purity (98.65%, 92.65%, and 85.5%) continuous and rapid separation of 5 μM, 10 μM, and 20 μM particles was achieved.

These studies demonstrate the broad applications of microfluidic technology in cell separation, particle classification, and high-throughput analysis. From the long-term culture and aging analysis of diploid yeast cells to the efficient separation of blood cells, stem cells, and tumor cells, different microfluidic chip designs have made significant progress in separation efficiency, purity, and resolution. The techniques employed, such as DLD arrays, contraction–expansion arrays, inertial sorting, and cascade sorting, not only improve sorting accuracy but also expand their potential in biomedical fields, especially in cancer research, stem cell therapy, and precision medicine. These advancements reflect the flexibility and adaptability of microfluidic technology, which can be tailored to optimize chip structures and separation mechanisms based on specific requirements, improving processing efficiency and ease of operation. As researchers, it is worth considering how to further enhance chip integration and automation, while exploring the multidimensional processing capabilities of more biological samples to promote the application of microfluidic technology in clinical diagnostics and personalized medicine.

### 3.2. High-Throughput and Parallelized Design

The design of multi-channel parallel working systems can significantly improve experimental efficiency by allowing the simultaneous processing of multiple samples or reactions, thereby reducing waiting time and experimental duration. This design not only enhances throughput and accuracy but also plays a crucial role in the high-throughput screening and precise control of microfluidic volumes. Studies have shown that multi-channel parallel microfluidic networks, built with the concept of column arrays, can further increase throughput, expanding the application prospects of microfluidic systems in clinical and chemical engineering fields [91]. Another study developed a scalable DLD-based microfluidic technology for isolating mesenchymal stem cells (MSCs), as shown in Figure 4f. This novel multi-chip DLD system consists of four parallel DLD sorting units, coupled with a hydrophobic coating, and can process 2.5 mL of raw bone marrow mononuclear cells (BMA) in 20 ± 5 min. Compared to traditional centrifugation methods, the system improves MSC recovery by two folds [86]. In the same year, Li et al. [92] proposed a multi-stage microfluidic method for CTC isolation. This method first sorts CTCs using a size-based dual-array DLD chip, then further purifies the mixed CTCs using a stiffness-based conical channel chip, and finally performs cell type identification using Raman spectroscopy.

The integration of column array concepts into multi-channel parallel microfluidic networks has been shown to significantly enhance device throughput, paving the way for broad applications in clinical and chemical engineering fields. Additionally, scalable DLD-based microfluidic systems have made breakthroughs in cell separation and MSC recovery, improving processing efficiency and recovery rates while demonstrating superior separation performance compared to traditional methods. Similarly, the multi-stage microfluidic sorting method, by combining different arrays and techniques, enables efficient CTC isolation and cell type identification.

## 4. Challenges in Microfluidic DLD-Based Cell Sorting

Despite the optimization of various geometries and arrangements (such as hexagonal-arranged triangular geometries and heart-shaped microstructures), further refinement is still required for complex mixed samples. For instance, in clinical applications, DLD technology is utilized for the separation and screening of CTCs. However, the heterogeneous nature of blood samples, containing cell populations with varying sizes and stiffness, results in a decline in separation efficiency as the system is scaled up. Additionally, issues such as cell aggregation and clogging further impact the performance of the system. Studies have shown that the separation efficiency of DLD technology is closely related to factors such as the shape, size, arrangement, angle, and density of the pillars in the array. Therefore, precisely controlling these parameters across varying particle sizes, shapes, and flow rates remains a significant technical challenge. In recent years, several emerging technologies have been introduced to the DLD field to improve device performance and address challenges associated with scaling up applications. For instance, advances in 3D printing and multi-layer soft lithography have enabled the more precise fabrication of complex DLD structures, thereby enhancing reproducibility [74]. Adjustable PNIPAM thermo-responsive hydrogel pillars can modulate pillar diameter through temperature control, facilitating the sorting of different particles and enhancing adaptability [75]. Flexible microfluidic materials, made from elastic polymers or biomimetic materials, help reduce shear forces, improve cell viability, and mitigate biocompatibility concerns [75]. Moreover, the integration of cascade inertial sorting with DLD leverages inertial effects to pre-enrich samples, reducing the load on the DLD device and improving sorting efficiency [90].

Despite the advancements in DLD sorting accuracy enabled by the aforementioned technologies, numerous challenges persist in high-throughput applications. Additionally, how to ensure the coordination and stable performance of each module in microfluidic chips that integrate multiple separation stages or modular designs remains a critical issue in practical applications. Optimizing the parallel design in multi-channel systems to handle different samples and reduce device complexity continues to be a significant challenge in improving high-throughput and parallel separation efficiency.

The diversity of cell types presents challenges due to differences in cell size, stiffness, and surface properties. Designing channels that accommodate various cell types to improve device throughput and particle separation efficiency, as well as separating micron and submicron particles and distinguishing particles of similar size or high concentration, remains challenging [36]. In high-throughput applications, cell aggregation and channel clogging represent significant bottlenecks in high-throughput DLD systems, impacting the long-term operational stability of the devices. Although DLD focusing theory has greatly improved, its implementation is still somewhat limited by clogging caused by particle–particle or particle–surface interactions [93]. The optimization of fluid dynamics and the use of surface anti-adhesion materials, such as superhydrophobic coatings, can help reduce particle deposition; however, further refinement is required. Anisotropic permeability, the inherent tendency of microfluidic arrays to cause unwanted lateral pressure gradients and unpredictable particle trajectories, can become particularly severe in arrays with unequal axial and lateral gaps or highly asymmetric shapes [94]. These non-uniform effects can be predicted and corrected through numerical simulations, such as CFD analysis, and optimized using machine learning techniques. Moreover, the fabrication process for DLD devices is quite complex. The high manufacturing costs of current DLD devices pose a significant barrier to the industrialization of microfluidic chips. By employing scalable fabrication techniques, such as roll-to-roll lithography, and implementing standardized designs, both cost reduction and improved usability can be achieved.

Future advancements should focus on integrating multiple separation stages to develop more efficient modular microfluidic chips, ensuring the coordinated operation of separation modules and enhancing system stability [90]. Optimizing multi-channel parallel designs will reduce device complexity [86], while AI algorithms can be employed to optimize fluid dynamics parameters and mitigate clogging issues. DLD technology shows significant potential in high-throughput applications, precision medicine, and biotechnology. However, further breakthroughs in material innovation, manufacturing processes, and fluid dynamics control are required to facilitate its industrialization and clinical translation.

## 5. The Current State of Research on the Application of Microfluidic DLD Technology

### 5.1. Biomedical Research

#### 5.1.1. Cancer

**Figure 5:**
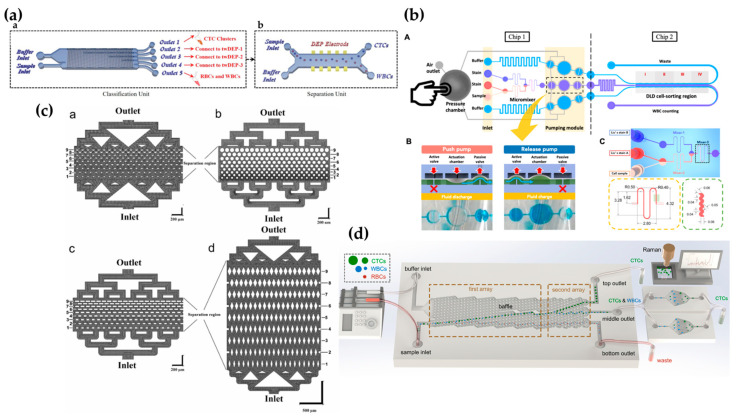
(**a**) An overview of the proposed hybrid microfluidic device for sorting CTCs and WBCs. **a** cascade DLD for size-based cell classification, **b** traveling-wave dielectrophoresis for cell separation. In the classification unit, CTCs and WBCs are categorized with a specific diameter, and in the separation unit, CTCs and WBCs are discriminated against based on electrical properties [97]. (**b**) The design and working principle of the finger-powered cell-sorting microsystem chip for cancer research applications. **A** The top view of finger-powered cell sorting microsystem chip and the main function of the individual component. **B** The operation principle of the finger-powered microfluidic pumping module. **C** A design highlight of the cell-staining area with three micromixers. The cells sample is first mixed with an equal proportion of Liu’s stain A through Mixer-2 [98]. (**c**) A schematic diagram of the micropillar array chip (MPA-Chip), displaying micropillars with triangular, circular, rectangular, and rhombic cross-sections. Schematic images of MPA-chip with micro-pillars with the triangle **a**, circle **b**, rectangle **c** and lozenge **d** cross-sections. The fluid flow enters the chip at the inlet and exits from the outlet. The black region indicates the fluid area and the white region shows the pillars and other solid parts of the chip. Every chip contains 9 rows of micro-pillars (horizontal in these images) in the separation region; the numbers next to each image indicate the row numbers of the micro-pillars. The gap between the pillars decreases from 50 lm in the first row to 10 lm in the last row [39]. (**d**) A schematic illustration of the multistage microfluidic sorting system based on size and stiffness. The DMC has a height of 50 μM and a gap of 32 μM [92].

It is reported that millions of people die from cancer every year, with cancer patients still accounting for a significant proportion of global annual deaths [95]. In biomedical research, the early detection and treatment of cancer have significant research importance. Various labeled and unlabeled techniques have been applied for the isolation of CTCs in early cancer detection and treatment, among which microfluidic DLD technology has gained particular attention. Liu et al. [96] developed a multi-marker microfluidic chip for enriching heterogeneous CTCs from peripheral blood samples from breast cancer patients. The chip significantly improved capture efficiency through the size-selective effects of the DLD array and the synergistic action of multivalent oligonucleotides. The results indicated that the developed multi-marker chip enhanced the capture efficiency of cell lines with both high and low expressions of epithelial cell adhesion molecules (ECAMs), and the functionalized DNA nanostructure chip could accurately capture CTCs with different phenotypes. Varmazyari et al. [97] introduced a label-free cell separation microfluidic platform (see Figure 5a), combining a cascaded DLD array with a traveling-wave dielectrophoresis (twDEP) system. In the cascaded DLD unit, CTC clusters and red blood cells were successfully separated from blood samples. Other white blood cells (WBCs) and CTCs were classified based on their diameter and connected to the twDEP unit to separate CTCs from white blood cells (and even cells of the same size). Numerical simulations showed that this method achieved a recovery rate close to 93%, significantly improving the separation efficiency of cells of similar and different sizes. Lee et al. [98] integrated a finger-driven actuator, DLD cell sorting system, and embedded micro-mixer (see Figure 5b) for cell staining operations. Experimental results demonstrated that the system achieved a purity of 97.23% and a recovery rate of 88.74% in less than one minute.

Researchers have developed an integrated microfluidic device (IMD) consisting of a spiral chip (using DFF technology) and a DLD chip for the rapid separation of gastric cancer (GC) cells from ascitic fluid and peritoneal lavage fluid. This device successfully isolated cancer cells from a simulated peritoneal lavage fluid containing 1/10,000 cancer cells and 12 complete GC ascitic fluid samples [99]. In a previous study, a new CTC capture method was introduced using the MPA-Chip (see Figure 5c), where the rhombic geometry achieved over an 85% capture efficiency, more than a 90% purity, and a 97% viability [39]. Additionally, the CTC sorting system developed by paralleling four DMC dual-array DLD chips (see Figure 5d) could process 2.5 mL of sample per minute, achieving a recovery rate of 96.30 ± 2.10% and a purity of 98.25 ± 2.48% [92]. This study also developed a sorting method based on a conical channel, utilizing solid and fluid dynamics coupling analysis. This method improved the separation efficiency of CTCs from white blood cells, increasing purity by 1.8 times. Huang et al. [83] designed an integrated microfluidic biochip for the separation of target tumor cells and their clusters from whole blood. This method used coating technology and incorporated a tumor cell suspension from 4T1 tumor-bearing BALB/c mice into the blood of the same mice for experimentation. The results showed that the DLD cluster sorting step was able to recover 88.58% of the tumor cell clusters with a purity of 92.20%, while the lateral filter in the bottom chip captured 89.54% of the tumor cells with a purity of 89.44%. To improve separation efficiency, the researchers optimized the fluid behavior around the walls.

Moreover, a study on the cascade microfluidic separation of tumor cells further enhanced the purity and separation efficiency of CTCs through sheathless inertial focusing in low-aspect-ratio spiral microchannels [89]. Another study pointed out a simple microfluidic method for the isolation of extracellular vesicles (EVs) using a micro-DLD array design, which has potential for separating nanobiological entities [100]. Mirhosseini et al. [101] introduced a microchannel design enhanced by boundary correction (BC). This improved microchannel effectively increased separation efficiency, with CTC throughput efficiency exceeding 93%, and more than 89% of tumor cells separated from the microfluidic channel outlet.

By combining DLD technology with other sorting techniques, such as twDEP, spiral chips, and conical channels, researchers have developed various efficient and scalable microfluidic platforms for achieving high recovery rates, purity, and separation efficiency for CTCs. Combining different microfluidic technologies, especially through multi-channel parallel systems and fluid dynamic optimization, can further improve separation accuracy and processing efficiency, driving cancer detection and treatment toward personalized and precise medicine. Future research can explore the application of these technologies in clinical settings, such as real-time monitoring, early screening, and treatment response evaluation, to address challenges in cancer treatment.

#### 5.1.2. Blood Analysis

**Figure 6:**
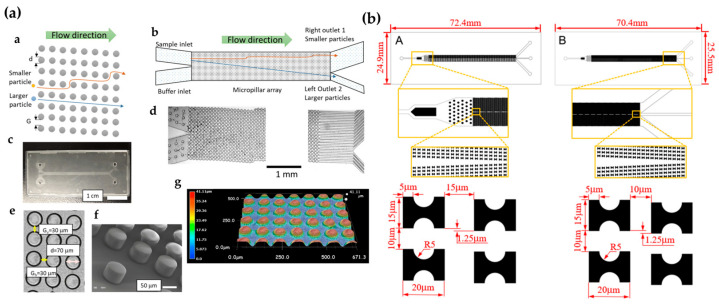
(**a**) **a** A schematic diagram of the DLD principle, **b** the layout of the microfluidic channels in the study, **c** a photograph of the polypropylene (PP)-based microfluidic DLD device, **d** microscopic images of the inlets and outlets of the microfluidic DLD device (Chip 1), **e** optical microscope images showing the dimensions and gaps of the micropillars, **f** scanning electron microscope (SEM) images of the micropillars, and **g** cross-sectional images from the 3D reconstruction [104]. (**b**) A CAD structure drawing and the size parameters of the chips. **A** and **B** are diagrams of chips with different lengths, widths, and micropillar geometries [66].

Blood is the most complex biofluid in the human body, consisting of various circulating cell types (such as red blood cells (RBCs), WBCs, platelets, and others) as well as biomolecules. Blood analysis is crucial for assessing the health status and disease conditions of patients [102]. Red blood cells are responsible for carrying oxygen and carbon dioxide, maintaining the body’s oxygen–carbon balance. In addition to immune cells, CTCs and other diseased cells are present in the blood during cancer metastasis, which is of significant importance for cancer prognosis, diagnosis, and treatment monitoring [103]. For example, the microfluidic chip inspired by fish gill structures, mentioned earlier, has been used to separate blood cells [81], as well as a method combining the cascade DLD array with the twDEP system for extracting CTCs and red blood cells from blood samples [97], both showing significant results. White blood cells are key components of the immune system, protecting the body against infections and diseases. Therefore, monitoring the levels of white and red blood cells is essential for evaluating the health of patients during treatment. Matsuura et al. [104] reported a polypropylene-based microfluidic DLD device (see Figure 6a) used for separating RBCs and WBCs from both bovine and human blood samples without the need for electronic devices. Experiments involving bovine blood cells showed that the device could effectively separate lymphocytes and neutrophils from diluted whole blood. In human blood samples, larger particles, such as neutrophils, could be collected by adjusting the Dc value. However, due to the similar diameter of human red blood cells and lymphocytes, separating human RBCs and WBCs using this device was more challenging than separating bovine blood cells. Nevertheless, due to the biocompatibility of polypropylene resin, this microfluidic DLD device has broad applications in blood cell collection, plasma exchange therapy, and clinical diagnostics.

Lv et al. [66] developed a DLD chip utilizing I-pillars (see Figure 6b) to classify RBCs with varying deformability. Through two-dimensional finite element simulations, they analyzed the interaction between the motion paths of different deformable RBC models and the internal flow field of the chip. The study revealed significant differences in the effect of fluid flow on RBCs with different deformabilities, with the flow field, micropillar structure, and RBC interactions determining the final trajectory of the RBCs. Chavez-Pineda et al. [105] introduced an innovative microfluidic platform that allows the separation and recovery of WBCs from diluted whole blood in a single step. This platform employs a novel sheathless method, initially precipitating blood cells and focusing them into a dense stream, followed by separation based on cell size using DLD technology. Experimental results showed that this platform successfully separated WBCs from diluted blood with a 100% separation efficiency, 76% recovery rate, and 80% purity, while maintaining a 98% cell viability. In the same year, another study successfully enhanced RBC separation efficiency (based on the ratio of RBCs separated at the lower exit of the microfluidic channel to all cells) to over 77% by improving the channel boundary structure [101].

DLD technology has made significant progress in blood cell separation and tumor cell extraction. For instance, microfluidic chips inspired by fish gill structures have been used for blood cell separation, while the combination of DLD and twDEP systems has been employed for circulating tumor cell extraction. Other studies have reported the use of polypropylene-based DLD devices and innovative sheathless methods, which show broad potential in separating RBCs and WBCs and in therapeutic and clinical diagnostic applications. However, separating blood cells remains challenging due to the morphological and flow characteristics of different cell types, necessitating further optimization of microfluidic devices and operational parameters to improve separation efficiency and cell recovery rates. These findings highlight the enormous potential of DLD technology in blood cell separation, particularly in tumor detection, immune monitoring, and the treatment of blood-related diseases.

#### 5.1.3. Single Cells and Stem Cells

Biomedical research plays a crucial role in improving human health, treating diseases, and advancing medical science. The first step in single-cell workflows is typically to separate heterogeneous cell suspensions into homogeneous components, which is essential for ensuring precise capture and analysis with microfluidic devices [106]. In particular, stem cell research has gained significant attention due to stem cells’ unique regenerative capabilities and their potential to differentiate into various cell types, making them a key component in disease treatment, regenerative medicine, and personalized healthcare. By effectively screening and classifying stem cells, we can not only deepen our understanding of their application potential in disease treatment but also provide more accurate treatment strategies for personalized medicine.

Microfluidic DLD technology provides strong support for the efficient and accurate screening of stem cells. By precisely controlling fluid dynamics, DLD technology allows for the accurate separation and enrichment of stem cells, laying a solid foundation for subsequent analysis and treatment. For example, a scalable microfluidic cascade sorter, previously mentioned, has been successfully applied to the high-throughput label-free enrichment of ADSCs from human adipose samples following tissue digestion. The study found that after cascade enrichment with the microfluidic sorter, the proliferation capacity of the ADSC subpopulations in tissue culture was increased by six times, marking a significant advancement for clinical cell therapy and regenerative medicine [88].

Additionally, a study in September 2023 successfully applied a novel multi-chip DLD system for the separation of MSCs. Compared to traditional centrifugation methods, this multi-chip DLD system increased the MSC recovery rate by two times, further validating the advantages and potential of microfluidic DLD technology in stem cell separation [86]. This technology enables scientists to perform more efficient and precise cell sorting and enrichment, providing reliable technical support for stem cell therapy, disease intervention, and personalized diagnostics.

Zhao et al. [99] developed a microfluidic chip utilizing Dean flow separation and DLD technology. This chip not only enables the high-throughput, rapid detection of label-free free GC cells in ascites and peritoneal lavage fluid but also allows single-cell analysis of cancer cells in ascites, thereby improving the diagnosis of peritoneal metastasis and providing important support for targeted therapy research.

Microfluidic DLD technology has shown immense potential not only in stem cell sorting but also in providing novel solutions for cell therapy, regenerative medicine, and personalized healthcare, opening new pathways for early disease diagnosis and precise treatment. Through microfluidic DLD technology, the separation efficiency of ADSCs and MSCs has been significantly enhanced, greatly promoting the progress of clinical cell therapy. Moreover, these technologies have also been applied to high-throughput cancer cell detection, offering new directions for the diagnosis of peritoneal metastasis.

### 5.2. Integration with Machine Learning

**Figure 7:**
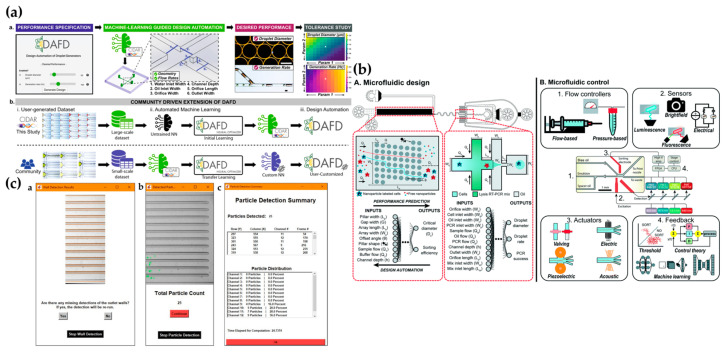
(**a**) The workflow of the developed design automation tool for flow-focusing droplet generators, referred to as DAFD. **a** The machine learning algorithms convert the user-specified performance into the required geometry and flow rates to achieve the desired droplet diameter and generation rate. The asterisk represents the tolerance value, which is the performance deviation. **b** Accurate predictive models (neural networks with CIDAR logo) trained on a large-scale data-set (initial learning) are made possible by machine learning and a low-cost rapid prototyping method that enabled efficient generation of a large-scale data-set in this study [108]. (**b**) An overview of machine learning-enabled automated microfluidic design and control. **A** Complex microfluidic devices, such as the MATE-seq platform, is comprised of two components, a deterministic-lateral-displacement array and droplet generator, which can be parameterized to describe both the physical design and experimental conditions. **B** Microfluidic devices, such as a droplet sorter, can consist of a series of vital non-fluidic modules [26]. (**c**) Particle detection results. **a** wall detection results, **b** particle detection results, **c** a detailed summary of the particle detection results with the particle distribution calculation [110].

Currently, research integrating machine learning primarily focuses on the application and optimization of microfluidic technologies. Examples include the microfluidic detection of live cell phenotypic biomarkers for cancer patient risk stratification using machine learning [107], the automated design of microfluidic droplet generation through machine learning [108] (see Figure 7a), and the combination of machine learning techniques with high-precision DLP printing [109]. In some intelligent systems, the training data for the network are entirely generated by the microfluidic platform itself. While there are limitations to self-collected data, they can undergo advanced training using networks based on previously trained processes from transfer learning techniques, leading to promising outcomes. As previously mentioned, machine learning for microfluidic device design and control (see Figure 7b) represents a cross-disciplinary integration that opens up new research directions and brings innovative possibilities to the field of cell sorting and separation.

Although DLD is a powerful tool and the development of high-throughput DLD separation devices holds great promise for diagnostics and treatment, much of the data analysis in DLD research still relies on error-prone and time-consuming manual processes. Emerging machine learning technologies can effectively assist in addressing the various challenges encountered in DLD experiments. Gao et al. [111] reported a machine learning-assisted microfluidic nanoplasma digital immunoassay technology to meet the growing demand for cytokine storm monitoring in COVID-19 patients. Compared to traditional manual counting methods, the convolutional neural network (CNN)-enhanced image processing approach not only greatly simplified the workflow but also reduced the processing time by 6000 times, while maintaining high statistical accuracy. This innovative method significantly improves the efficiency and precision of cytokine monitoring, providing a more efficient tool for the clinical diagnosis and treatment of COVID-19. Gioe et al. [110] developed a reliable particle detection method (see Figure 7c) as the foundation for DLD separation analysis. The results showed that this method drastically shortened the video analysis time during the DLD separation process, achieving a particle detection accuracy of 97.86%, with an average computational time of only 25.274 s. Machine learning-assisted microfluidic DLD research is both a current trend and an important direction for future development.

At present, many studies are still in the early exploration phase and face the gap between theory and practice. While the application of machine learning models in microfluidic DLD shows promise, there are still limitations in channel design, fluid dynamics simulations, and data analysis accuracy. Furthermore, the efficiency and operability of microfluidic DLD technology need further optimization, particularly in ensuring reproducibility and stability during large-scale applications. These challenges still need to be addressed. Therefore, the integration of machine learning with microfluidic DLD technology has not yet reached its full potential and requires more interdisciplinary collaboration and technological innovation to advance this field deeply. In image classification tasks, CNNs have become the most popular algorithm for cell classification due to their outstanding performance in deep learning. The advantage of using CNNs for cell classification lies in their ability to automatically perform feature extraction and encapsulation, eliminating the need for manual intervention in specific feature processing tasks [112]. Specifically, microfluidic chips are used to capture cells and perform initial separation based on their physical characteristics (such as size, shape, rigidity, etc.), while the CNN model is responsible for further classifying the separated cell images. The classification results generated by the CNN model are fed back into the experimental system, enabling real-time adjustments to the operations of the microfluidic chip to optimize sorting accuracy. To achieve this integration, appropriate hardware configurations, such as Dell XPS 15, Equipped with an Intel Core i7-9750H processor, GPUs (NVIDIA GeForce GTX 1650), and memory, are typically required [113]. In one study, Chen et al. [114] developed an optomechanical scanning imaging method, utilizing a field-programmable gate array (FPGA) to reconstruct cell images, which were then transmitted to the central processing unit (CPU) for feature extraction and cell classification. In terms of hardware integration, microfluidic chips capture cell images using high-speed cameras (e.g., FLIR black-and-white CMOS cameras). One study integrated mobile lensless microscopy with deep learning to automatically detect high-sensitivity C-reactive protein levels in human serum samples [115]. The camera transmits the image data in real time to the CPU via a USB 3.0 interface. The CNN model in the computer receives and processes this data, extracting image features and performing cell classification. Data exchange between the microfluidic chip and the CNN model occurs via TCP/IP or USB data transmission protocols. The classification results (e.g., labels) are then sent back to the experimental system, enabling adjustments to the microfluidic chip’s separation parameters based on the classification outcomes, thereby optimizing the cell sorting process.

To enhance the model’s learning ability and generalization performance, some studies preprocess the collected datasets (e.g., cell image datasets) by resizing, normalizing, and applying data augmentation techniques [116,117]. Through multiple convolutional layers, CNNs automatically extract hierarchical features from the images, allowing for the recognition of cell morphology, size, and other key biological characteristics. During model training, optimization parameters (such as the learning rate, batch size, and optimizer) are adjusted to continuously improve the model’s accuracy and robustness. Finally, cross-validation and validation set methods are employed to assess the model’s performance, using metrics such as accuracy, recall, and the F1 score to evaluate the effectiveness of the model in cell classification tasks.

However, many other algorithms, such as support vector machines (SVMs), logistic regression (LR), variational autoencoders (VAEs), decision trees, linear discriminant analysis (LDA), principal component analysis (PCA), and quadratic discriminant analysis (QDA), are widely used in the design and optimization of cell classification in intelligent microfluidic systems [118]. Although the CNN, as a leading deep learning algorithm, has demonstrated significant advantages in cell classification, its performance varies across different sample environments, especially when handling different types of cell classification tasks. The accuracy and robustness of a CNN depend on the specific context. In contrast, traditional classifiers like SVMs and LDA perform well in specific scenarios. For instance, SVMs excel in high-dimensional, small-sample data, particularly when the data are linearly separable, offering high precision and generalization ability [119]. LR [120] is well-suited for datasets with strong linear relationships, though its capacity to handle complex data is limited. LDA [121] is effective when there are clear distinctions between data features and the categories are linearly separable, improving classification accuracy. However, CNNs excel in automatic feature extraction and deep learning, especially when dealing with complex, noisy, or distorted image data. The key architectural components of CNNs, such as convolutional layers, pooling layers, and activation functions, enable efficient feature extraction and optimization, enhancing robustness against noise and interference. Pooling layers reduce data dimensionality through downsampling while preserving critical features, further boosting model resilience. Additionally, data augmentation techniques, including random cropping, rotation, and color jittering, contribute to CNNs’ ability to generalize well across diverse imaging conditions. However, the training process for CNNs typically requires large datasets and substantial computational resources, and their robustness may be compromised when dealing with small sample sizes or low-quality images. Based on the discussion above, this study provides a comprehensive comparison of these algorithms (see Table 2). By comparing the strengths and weaknesses of SVMs, LR, LDA, and CNNs, we can identify the most appropriate algorithm for specific cell classification tasks. Despite the progress made in combining microfluidic DLD technology and machine learning, challenges remain, particularly in the integration of theory and practice, the precision of fluid dynamics simulations, and ensuring stability and reproducibility in large-scale applications.

## 6. Conclusions and Outlook

Various optimization strategies for geometries and configurations, such as hexagonal arrangements, triangular geometries, and heart-shaped micropillar designs, have been proposed. Research indicates that combining adjustable PNIPAM thermoresponsive hydrogel pillars or cascaded inertial sorting with DLD technology can enhance separation efficiency. Additionally, multi-channel parallel systems have significantly improved experimental efficiency and throughput. In recent years, microfluidic chips based on DLD arrays have been developed for the efficient capture of CTCs with different phenotypes, demonstrating promising applications in cancers such as breast and gastric cancer. By integrating cascaded DLD arrays with traveling-wave dielectrophoresis, the separation efficiency and recovery rate of cancer cells and red blood cells have been further improved.

In blood analysis, DLD technology effectively separates various blood cell types, especially red and white blood cells, and is widely applied in blood cell separation, plasma exchange therapy, and clinical diagnostics. Particularly in CTC detection, DLD technology offers a label-free, rapid, and efficient sorting method. The application of DLD technology in biomedical research has also expanded, especially in stem cell screening and enrichment. By precisely controlling fluid dynamics, DLD provides strong support for stem cell separation, significantly enhancing cell recovery and proliferation, thus advancing cell therapy and regenerative medicine.

With the introduction of machine learning, the application prospects of DLD technology in automation, accuracy, and high-throughput processes have broadened. Machine learning plays a crucial role in image processing and data analysis, where CNN-assisted image processing has improved cytokine monitoring efficiency, shortened operation times, and significantly enhanced the accuracy of cell detection and particle recognition.

In the future, microfluidic DLD technology holds significant promise in personalized medicine, precision medicine, and early disease diagnosis. As precision medicine advances, DLD cell sorting technology is poised to play a pivotal role in cancer liquid biopsy, personalized drug screening, and immunotherapy, particularly in applications such as CTC screening, immune cell sorting, and stem cell sorting. For example, the multi-stage microfluidic CTC sorting method based on DLD enables the high-purity, high-throughput separation of CTCs, with a recovery efficiency of 96.30% ± 2.10% and a purity of 98.25% ± 2.48% [92]. Furthermore, DLD technology can be integrated with biosensors (such as fluorescence sensors and electrochemical sensors), microfluidic chips, and intelligent detection platforms, facilitating real-time monitoring and automated analysis, thereby enhancing diagnostic accessibility and efficiency. This is especially beneficial in resource-limited settings, where it can aid in blood analysis or infectious disease screening. For instance, integrating DLD technology into microfluidic biosensor platforms can enable point-of-care testing (POCT), improving the accessibility and efficiency of diagnostics, particularly in regions with limited resources for blood analysis or infectious disease screening.

However, to achieve widespread adoption, it is crucial to optimize existing designs, including enhancing separation efficiency, improving cell sorting capabilities, reducing operational complexity, and increasing throughput. Currently, DLD channel designs primarily rely on experimental data analysis or simulation tools (such as finite element methods) for modeling and validation. However, these methods have limitations in precisely controlling fluid dynamics and optimizing chip designs at a deeper level, often requiring extensive experimental procedures. In the coming years, research needs to break through traditional design concepts to develop more efficient microfluidic DLD solutions. For example, intelligent DLD designs that integrate artificial intelligence and machine learning to optimize structural configurations can enhance design precision and adaptability [108]. Moreover, combining computational fluid dynamics with deep learning can facilitate the automation of DLD design [125]. High throughput and clinical translation remain central to the development of DLD technology, particularly in the creation of portable devices with excellent biocompatibility. The utilization of novel materials and advanced manufacturing processes will further advance the application of POCT in personalized medicine. Additionally, DLD technology can be integrated with single-cell sequencing [126], mass spectrometry [127], and AI-based diagnostic systems [128], enabling high-throughput and high-precision disease detection. Nevertheless, several challenges are anticipated in the future, including data privacy and security concerns associated with AI and machine learning, compatibility issues in multi-technology integration, the interpretability of these advanced technologies [129], the reliability of novel materials and manufacturing processes [75,79], and the complexity of clinical translation [130].

In this context, we propose an innovative approach: using machine learning techniques to reverse-engineer the geometric structures and fluid dynamics of DLD channels. By incorporating deep learning and reinforcement learning, machine learning can extract patterns from experimental data and predict how different designs influence fluid behavior and separation efficiency. The integration of machine learning facilitates efficient automation, real-time processing, and a reduction in the number of experimental trials, leading to more intelligent and precise channel designs. This idea is highly feasible. As this concept evolves, it is expected to be industrialized in the future, potentially leading to groundbreaking advancements in healthcare and disease prevention.

## Figures and Tables

**Table 1:** The influence of channel geometry design on particle separation.

Geometry	Effect	Reference
Channel width	Lateral displacement occurrence	[31]
Channel height	Fluid flow velocity	[32]
Channel length	Particle flow time	[33]
Pillar size and spacing	Lateral displacement occurrence	[21,34]
Micropillar geometry	Clogging effects	[35,36]
Micropillar layout	Flow path, lateral displacement	[37]
Channel curvature	Fluid flow characteristics	[38]

**Table 2:** Comparison of machine learning classification algorithms.

Algorithm	Advantages	Limitations	Suitable Applications
CNN [116,117]	Capable of handling image data and automatically extracting features, with strong feature learning ability.	Requires a large amount of data for training, high computational cost, and long training times, with high data quality requirements.	Suitable for image processing, video analysis, and large-scale datasets.
SVM [119,122]	Strong classification performance and effective at handling nonlinear problems.	Sensitive to hyperparameter tuning, with slow training on large datasets and difficulty in handling large-scale data.	Suitable for small-sample, high-dimensional datasets, medical image classification, and diagnostic purposes.
LR [120]	Simple to compute and easy to implement, with strong model interpretability.	Limited in handling nonlinear relationships and prone to overfitting.	Suitable for linear classification tasks and models with fewer features and used in medical data and simple tasks.
VAE [113]	Handles complex data distributions and captures the underlying diversity of the data.	Long training times, high requirements for data preprocessing, and poor interpretability.	Suitable for modeling complex data distributions, generative models, unsupervised learning, and multimodal data.
Decision Tree [113]	Fast model training and easy to understand and interpret.	Poor interpretability in complex cases and susceptible to noise and missing data.	Suitable for low-dimensional, efficient feature classification, especially when dealing with smaller datasets.
LDA [113,121]	Effective at handling linearly separable data and simple to compute and implement.	Requires linear separability and has limited dimensionality reduction capabilities.	Suitable for high-dimensional, linearly separable datasets, such as in biomedical data analysis.
PCA [113,123]	Reduces computational complexity and helps extract important features from high-dimensional data.	Cannot be used for feature selection, has poor interpretability, is sensitive to outliers, and requires extensive data preprocessing.	Suitable for dimensionality reduction, data compression, and feature extraction in high-dimensional data, used in pattern recognition.
QDA [113,124]	Strong at handling nonlinear problems, with good generalization ability and no need for regularization.	High computational complexity, many parameters, and strict assumptions about data distribution.	Suitable for binary classification tasks, especially for small-sample data, such as in disease prediction.

## Data Availability

Not applicable.
